# (5,6-Dimethyl-1,10-phenanthroline)(2-{[2-(di­phenyl­phosphan­yl)benzyl­idene]amino}­ethan-1-amine)­platinum(II) dinitrate methanol disolvate

**DOI:** 10.1107/S2056989025001847

**Published:** 2025-03-04

**Authors:** Ashley Jurisinec, Yingjie Zhang, Janice R. Aldrich-Wright

**Affiliations:** a School of Science, Western Sydney University, Locked Bag 1797, Penrith South DC, Sydney, NSW 2751, Australia; b Australian Nuclear Science and Technology Organisation, Kirrawee DC, New South Wales, Australia; Universidad de la Repüblica, Uruguay

**Keywords:** crystal structure, platinum(II), tri­phenyl­phosphine, π-stacking

## Abstract

The title compound, [Pt(C_14_H_12_N_2_)(C_21_H_21_N_2_P)](NO_3_)_2_·2CH_3_OH, is a platinum(II) complex, which crystallizes in a monoclinic (*P*2_1_/*c*) space group. The complex exhibits a distorted square-planar geometry, which includes a monodentate 5,6-dimethyl-1,10-phenanthroline ligand and a tridentate 2-{[2-(di­phenyl­phosphan­yl)benzyl­idene]amino}­ethan-1-amine ligand. The structure reveals both intra- and inter­molecular π-stacking inter­actions between the phenanthroline and phosphine rings. Hydrogen bonding is observed between the complex ion, nitrate counter-ions and solvent mol­ecules.

## Abstract 2

The title platinum(II) complex exhibits a distorted square-planar geometry. Intra- and inter­molecular π-stacking inter­actions are observed between the phenanthroline and phosphine rings while hydrogen bonding is observed between the complex ion, nitrate counter-ions and solvent mol­ecules.

## 1. Chemical context

Platinum(II) complexes of the structure [Pt(A_L_)(H_L_)] have shown promise as potent chemotherapeutic agents (Kemp *et al.*, 2007 ▸). To further enhance this class of complexes, various approaches have been undertaken including oxidation to platinum(IV) and subsequent modification by the coordination of various bioactive and non-bioactive ligands in the axial positions (Khoury *et al.*, 2022 ▸). When investigating potential axial linking strategies, we discovered an unusual reaction between 2-(di­phenyl­phosphino)benzaldehyde and [Pt(5,6-dimethyl-1,10-phenanthroline)(1,2-di­amino­ethane)]^2+^ resulting in a novel coordination sphere. Typically, 1,10-phenanthroline and its derivatives coordinate as a bidentate ligand, however in this instance one of the Pt—N bonds is displaced by the introduction of 2-(di­phenyl­phosphino)benzaldehyde, resulting in a Pt—P bond and the formation of an imine with one of the 1,2-di­amino­ethane amines. Herein, we present the crystal structure of the title platinum complex.
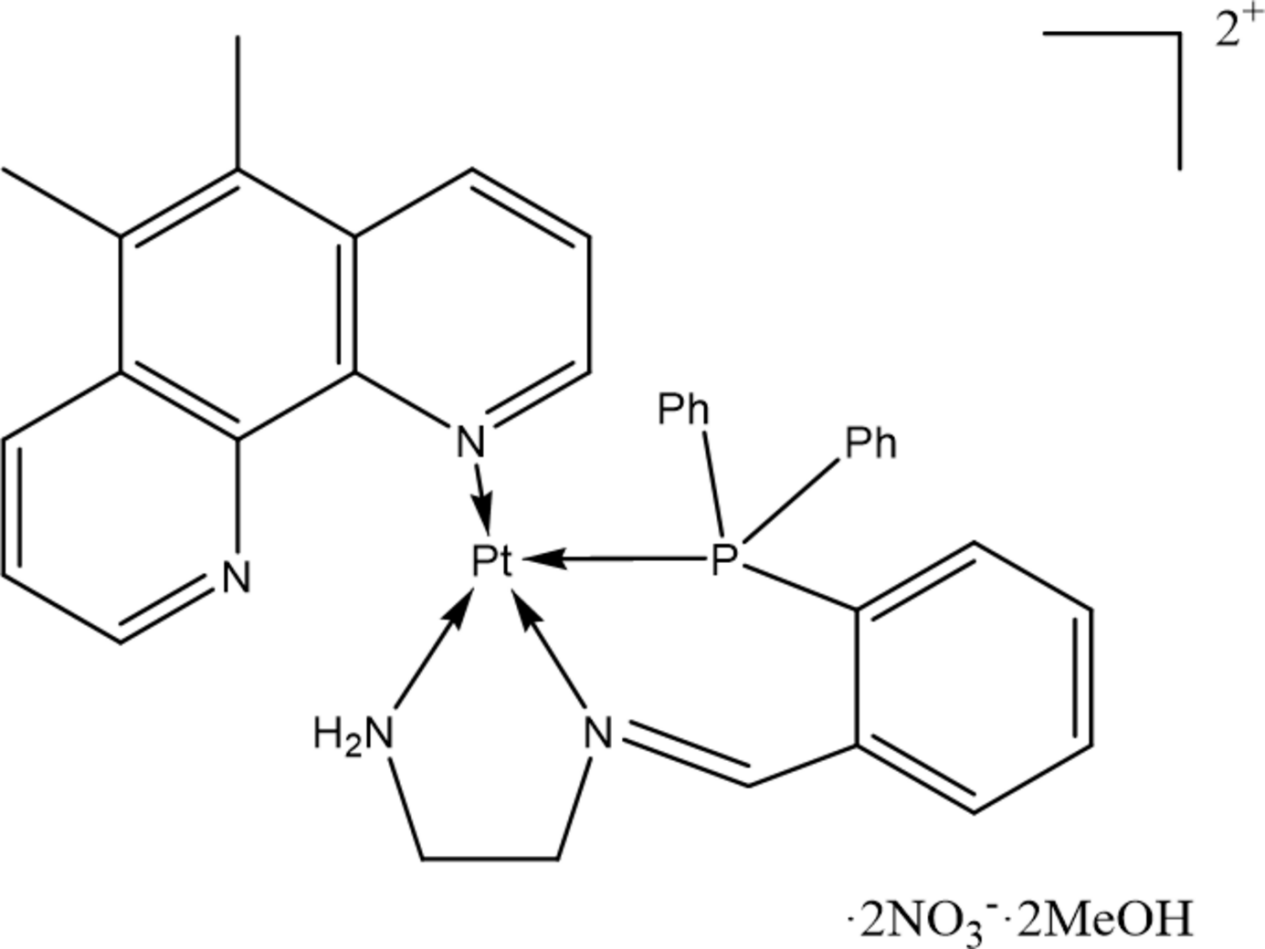


## 2. Structural commentary

**Figure 1:**
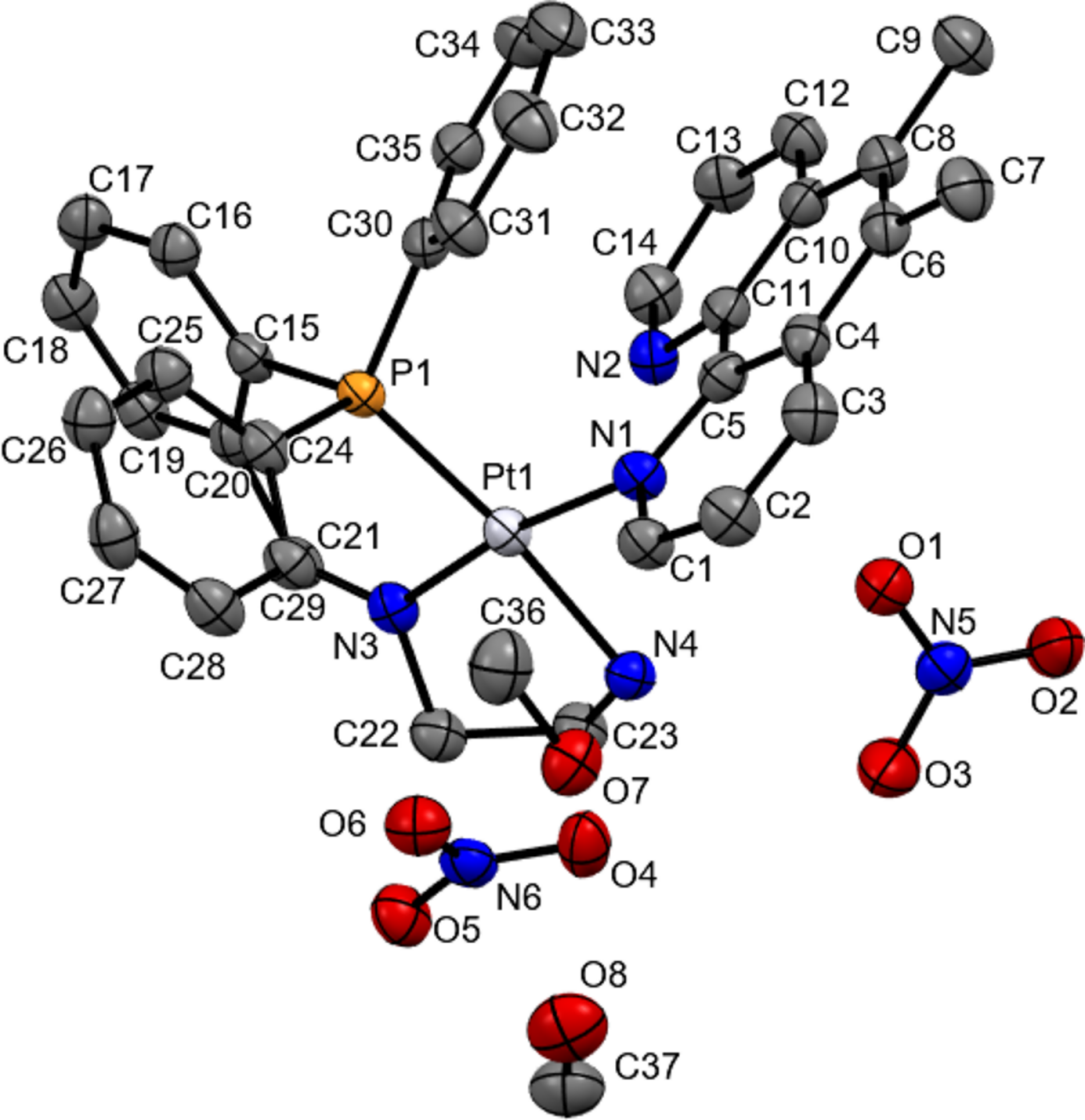
View of the title complex with atom-numbering scheme. Displacement ellipsoids are drawn at the 50% level.

**Figure 2:**
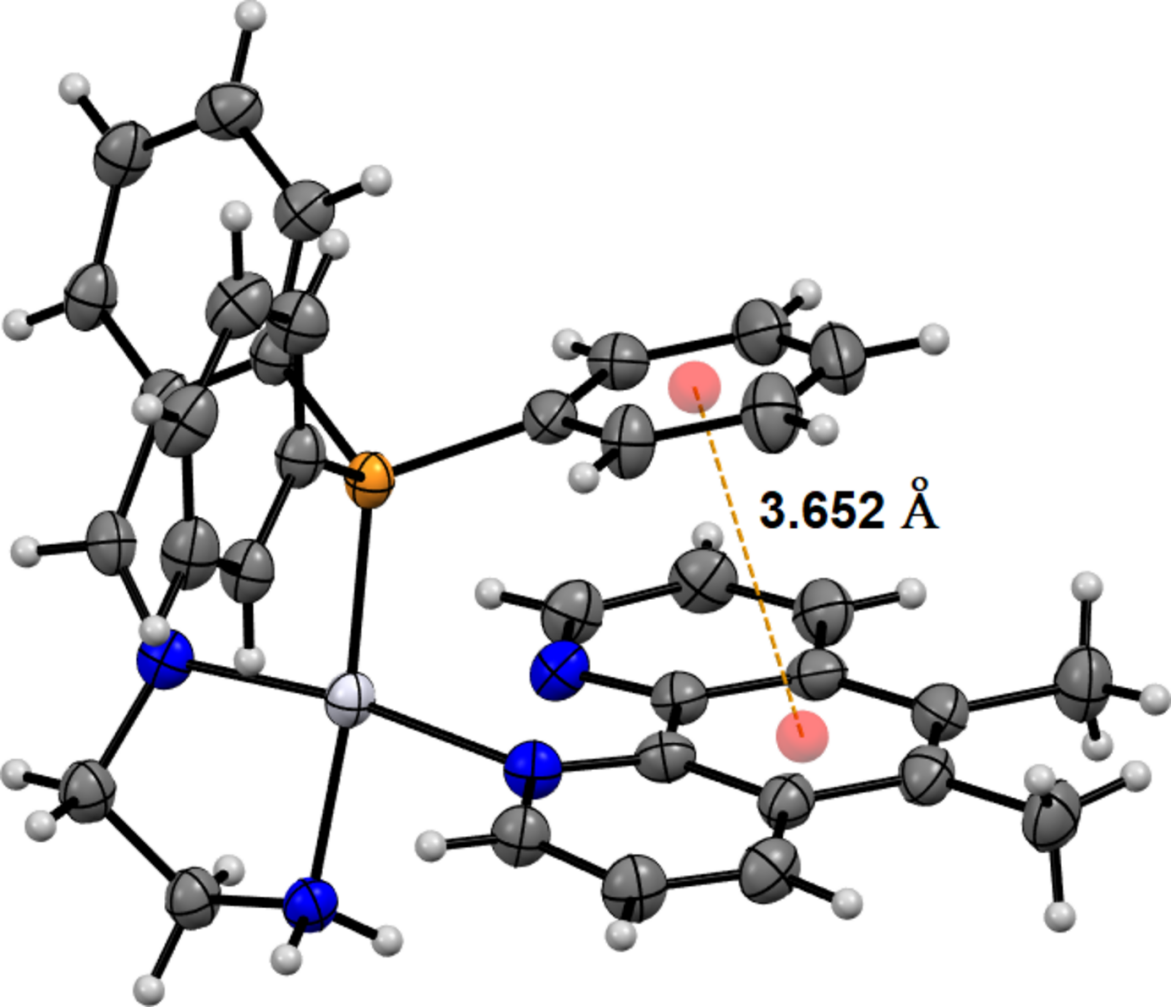
The intra­molecular π–π stacking inter­action.

The title complex contains a Pt^II^ ion coordinated with a monodentate 5,6-dimethyl-1,10-phenanthroline and a tridentate 2-{[2-(di­phenyl­phosphan­yl)benzyl­idene]amino}­ethan-1-amine ligand, with two nitrate counter-ions and two methanol solvent mol­ecules (Fig. 1 ▸). The Pt coordination exhibits a distorted square-planar geometry with the platinum atom sitting 0.0063 (13)#7emsp14;Å from the plane. The 5,6-dimethyl-1,10-phenanthroline ligand is coordinated through a single nitro­gen, with the extended ring structure sitting orthogonal to the coordinated amines. The centre of the phenanthroline ring sits below one of the tri­phenyl­phosphine rings, with a centroid–centroid distance of 3.652 (3) Å (Fig. 2 ▸), evidencing π-stacking inter­actions. The intra­molecular π–π stacking inter­action is quite unique as no similar case has been reported for Pt complexes with monodentate phenanthroline type ligands in CSD. Therefore, it is not essential for stabilizing the monodentate nature of phenanthroline ligands. The bond length for Pt—N1 [Pt—Phen, 2.052 (3) Å] is comparable to those in complexes bearing a monodentate 2,9-dimethyl-1,10-phenanthroline (Fanizzi *et al.*, 1994 ▸, 2004 ▸), and is also comparable to other bidentate examples (Kato & Takahashi, 1999 ▸; Brodie *et al.*, 2006 ▸). Despite imine derivatization of the 1,2-di­amino­ethane, the N3—Pt1—N4 bond angle is 83.30 (14)°, which is consistent with previously reported complexes with unmodified di­amines (Ellis & Hambley, 1994 ▸; Kato & Takahashi, 1999 ▸; Brodie *et al.*, 2006 ▸); however, the Pt—N4 (NH_2_) and Pt—N3 (N) bond lengths do differ. The Pt—N4 bond is 2.092 (4) Å, which is slightly longer than the prior di­amine examples which range from 2.031 to 2.044 Å for the Pt—NH_2_ bonds (Ellis & Hambley, 1994 ▸; Kato & Takahashi, 1999 ▸; Brodie *et al.*, 2006 ▸), whereas the Pt—N3 bond is slightly shorter at 1.984 (3) Å. These differences are attributed to the presence of the imine double bond. The nitrate counter-ions are located next to the NH_2_ with hydrogen-bonding inter­actions evident between the amine and the counter-ions (Table 1 ▸).

## 3. Supra­molecular features

**Figure 3:**
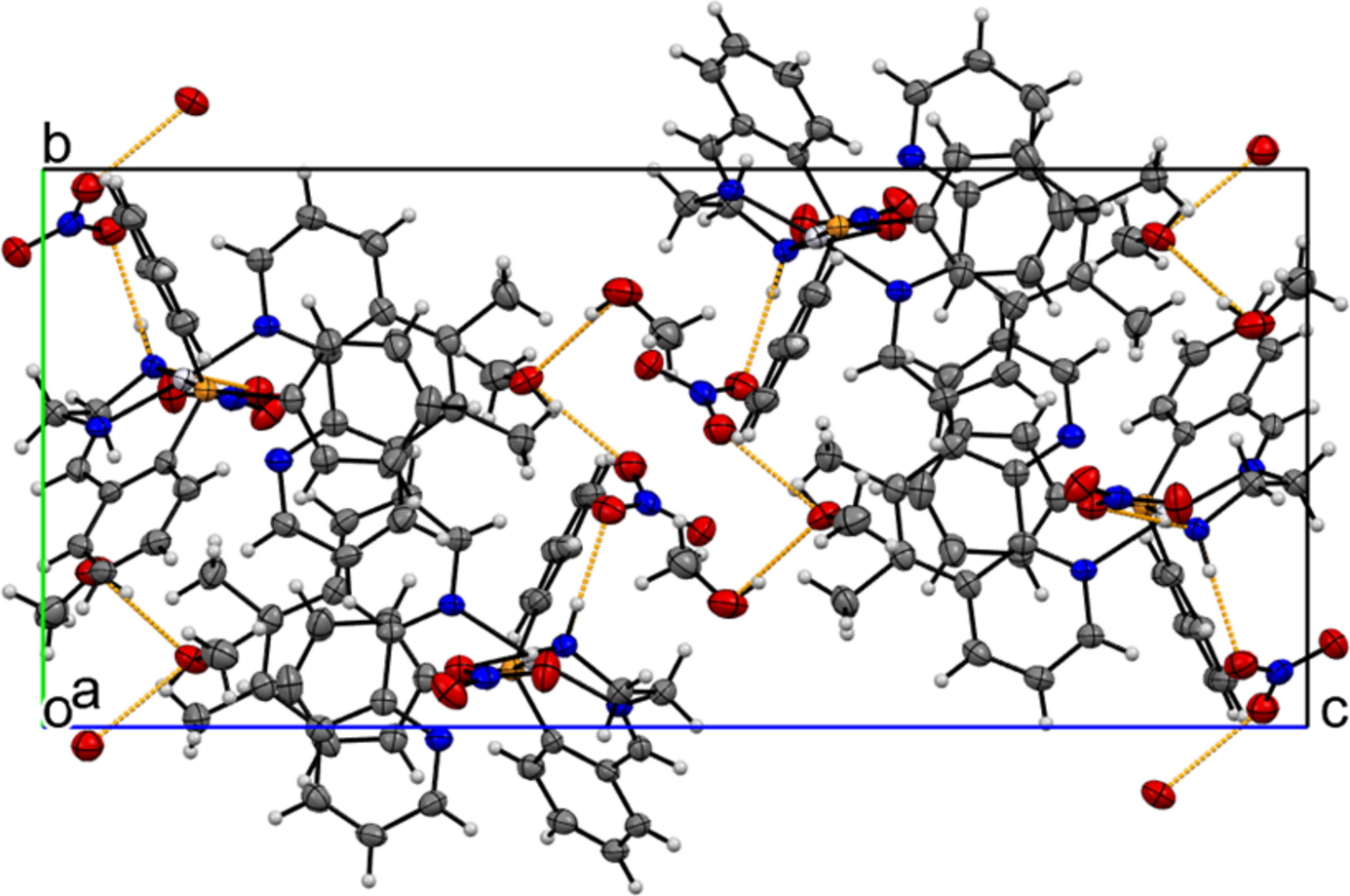
Crystal packing of the title complex with hydrogen-bonding inter­actions shown as dashed lines.

The complex mol­ecules are arranged in an inverted pattern, allowing for offset π-stacking inter­actions between the phenanthroline ligands [centroid–centroid distance = 4.179 (5) Å]. Hydrogen bonding is observed among the nitrate counter-ions and methanol solvent mol­ecules, with the solvent occupying the spaces between complex mol­ecules (Table 1 ▸, Fig. 3 ▸).

## 4. Database survey

Although 330 crystal structures were reported in the CSD (2022.3.0; Groom *et al.*, 2016 ▸) for Pt complexes involving phenanthroline ligands, Pt complexes with monodentate phenanthroline type ligands are rather rare. So far only eleven such structures were reported, almost all involving additional simple ligands, with the monodentate Pt—N bond lengths ranging from 2.037 to 2.181 Å. Among these eleven structures, nine include a monodentate 2,9-dimethyl-1,10-phenanthroline unit where the methyl groups are purported to induce steric effects, preventing bidentate coordination. Three of these structures [CSD Refcodes SOYZAH (Fanizzi *et al.*, 1992 ▸), POFJOJ and POFJUP (Fanizzi *et al.*, 1994 ▸)] also contain tri­phenyl­phosphine-type ligands, with monodentate Pt—N bond lengths of 2.181, 2.046 and 2.069 Å, respectively. Of the two structures that include a monodentate 1,10-phenathroline, one includes two tri­ethyl­phosphine and one chlorido ligands (CPEUPT; Bushnell *et al.*, 1974 ▸), whereas the other includes three penta­fluoro­benzene ligands (ZAXXOL; Usón *et al.*, 1995 ▸) with monodentate Pt—N bond lengths of 2.136 and 2.140 Å respectively.

## 5. Synthesis and crystallization

The synthesis of the title complex was achieved *via* reaction between [Pt(5,6-dimethyl-1,10-phenanthroline)(1,2-di­amino­ethane)](NO_3_)_2_ (Pt56MEEN) and 2-(di­phenyl­phosphino)benzaldehyde. Pt56MEEN was synthesized as its chloride salt using a modified literature method (Brodie *et al.*, 2004 ▸). First, 1,2-di­amino­ethane was reacted with an equimolar amount of K_2_PtCl_4_ in water. The resultant yellow precipitate was refluxed with 5,6-dimethyl-1,10-phenanthroline to yield a pale-yellow solution. The complex was then isolated using reverse-phase C18 chromatography and converted to its nitrate salt by addition of two molar equivalents of AgNO_3_ in water. The AgCl precipitate was removed by vacuum filtration and the solution dried. The complex was suspended in methanol and stirred with 1.25 molar equivalents of 2-(di­phenyl­phosphino-benzaldehyde) at 323 K overnight to form a clear orange solution. The desired product was isolated using reverse-phase C18 flash chromatography with a water–methanol mobile phase. Crystals of the title complex were formed using vapour diffusion of diethyl ether into a solution of the complex in methanol.

## 6. Refinement

Crystal data, data collection and structure refinement details are summarized in Table 2 ▸. The single-crystal data were collected at 100 (2) K on the MX1 beamline (Cowieson *et al.*, 2015 ▸) at the Australian Synchrotron employing silicon double crystal monochromated synchrotron radiation (λ = 0.71078 Å). Hydrogen atoms were added to the calculated positions and refined using a riding model. Potential hydrogen bonds were calculated using *PLATON* (Spek, 2020 ▸).

## Supplementary Material

Crystal structure: contains datablock(s) I. DOI: 10.1107/S2056989025001847/ny2011sup1.cif

Structure factors: contains datablock(s) I. DOI: 10.1107/S2056989025001847/ny2011Isup2.hkl

Supporting information file. DOI: 10.1107/S2056989025001847/ny2011Isup3.cdx

CCDC reference: 2427287

Additional supporting information:  crystallographic information; 3D view; checkCIF report

## Figures and Tables

**Table 1:** Hydrogen-bond geometry (Å, °)

*D*—H⋯*A*	*D*—H	H⋯*A*	*D*⋯*A*	*D*—H⋯*A*
N4—H4*A*⋯O1	0.91	1.96	2.872 (5)	176
N4—H4*B*⋯O4	0.91	2.05	2.920 (5)	159
O7—H7⋯O6	0.84	1.96	2.771 (5)	162
O8—H8⋯O7	0.84	2.04	2.872 (5)	170
C3—H3⋯O1^i^	0.95	2.36	3.244 (6)	154
C3—H3⋯O2^i^	0.95	2.57	3.168 (6)	121
C9—H9*A*⋯O4^ii^	0.98	2.48	3.235 (6)	134
C9—H9*B*⋯O5^iii^	0.98	2.53	3.494 (6)	170
C14—H14⋯O7^iv^	0.95	2.57	3.417 (6)	149
C18—H18⋯O5^v^	0.95	2.58	3.288 (6)	131
C22—H22*A*⋯O3^vi^	0.99	2.47	3.422 (6)	162
C22—H22*B*⋯O5	0.99	2.44	3.385 (6)	160
C25—H25⋯O2^vii^	0.95	2.40	3.282 (5)	154
C27—H27⋯O6^viii^	0.95	2.58	3.278 (6)	131
C29—H29⋯O6	0.95	2.40	3.094 (5)	130

Symmetry codes: (i) 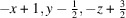; (ii) 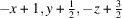; (iii) 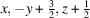; (iv) 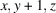; (v) 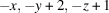; (vi) 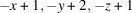; (vii) 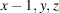; (viii) 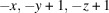.

**Table 2:** Experimental details

Crystal data	
Chemical formula	[Pt(C_14_H_12_N_2_)(C_21_H_21_N_2_P)](NO_3_)_2_·2CH_4_O
*M*_r_	923.82
Crystal system, space group	Monoclinic, *P*2_1_/*c*
Temperature (K)	100
*a*, *b*, *c* (Å)	12.692 (3), 11.349 (2), 25.812 (5)
β (°)	95.29 (3)
*V* (Å^3^)	3702.1 (13)
*Z*	4
Radiation type	Silicon double crystal monochromated synchrotron, λ = 0.71078 Å
μ (mm^−1^)	3.89
Crystal size (mm)	0.01 × 0.01 × 0.004

Data collection	
Diffractometer	Eiger Detector
Absorption correction	Multi-scan (*SADABS*; Krause *et al.*, 2015 ▸)
*T*_min_, *T*_max_	0.339, 0.431
No. of measured, independent and observed [*I* > 2σ(*I*)] reflections	46431, 7437, 6419
*R*_int_	0.109
(sin θ/λ)_max_ (Å^−1^)	0.673

Refinement	
*R*[*F*^2^ > 2σ(*F*^2^)], *wR*(*F*^2^), *S*	0.040, 0.108, 1.04
No. of reflections	7437
No. of parameters	484
No. of restraints	1
H-atom treatment	H-atom parameters constrained
Δρ_max_, Δρ_min_ (e Å^−3^)	2.77, −1.38

Computer programs: *XDS* (Kabsch, 1993 ▸, 2010 ▸), *BlueIce* (McPhillips *et al.*, 2002 ▸), *SHELXT2018/2* (Sheldrick, 2015*b* ▸), *SHELXL2018/3* (Sheldrick, 2015*a* ▸) and *OLEX2* (Dolomanov *et al.*, 2009 ▸).

## supplementary crystallographic information

### (5,6-Dimethyl-1,10-phenanthroline)(2-{[2-(diphenylphosphanyl)benzylidene]amino}ethan-1-amine)platinum(II) dinitrate methanol disolvate Crystal data

**Table:**

[Pt(C_14_H_12_N_2_)(C_21_H_21_N_2_P)](NO_3_)_2_·2CH_4_O	*F*(000) = 1848
*M**_r_* = 923.82	*D*_x_ = 1.657 Mg m^−^^3^
Monoclinic, *P*2_1_/*c*	Silicon double crystal monochromated synchrotron radiation, λ = 0.71078 Å
*a* = 12.692 (3) Å	Cell parameters from 12000 reflections
*b* = 11.349 (2) Å	θ = 1.6–28.6°
*c* = 25.812 (5) Å	µ = 3.89 mm^−^^1^
β = 95.29 (3)°	*T* = 100 K
*V* = 3702.1 (13) Å^3^	Block, yellow
*Z* = 4	0.01 × 0.01 × 0.004 mm

### (5,6-Dimethyl-1,10-phenanthroline)(2-{[2-(diphenylphosphanyl)benzylidene]amino}ethan-1-amine)platinum(II) dinitrate methanol disolvate Data collection

**Table:**

Eiger Detector diffractometer	6419 reflections with *I* > 2σ(*I*)
Radiation source: Australian Synchrotron MX1	*R*_int_ = 0.109
π scans	θ_max_ = 28.6°, θ_min_ = 1.6°
Absorption correction: multi-scan (SADABS; Krause *et al.*, 2015)	*h* = −13→14
*T*_min_ = 0.339, *T*_max_ = 0.431	*k* = −13→13
46431 measured reflections	*l* = −32→32
7437 independent reflections	

### (5,6-Dimethyl-1,10-phenanthroline)(2-{[2-(diphenylphosphanyl)benzylidene]amino}ethan-1-amine)platinum(II) dinitrate methanol disolvate Refinement

**Table:**

Refinement on *F*^2^	1 restraint
Least-squares matrix: full	Hydrogen site location: inferred from neighbouring sites
*R*[*F*^2^ > 2σ(*F*^2^)] = 0.040	H-atom parameters constrained
*wR*(*F*^2^) = 0.108	*w* = 1/[σ^2^(*F*_o_^2^) + (0.0607*P*)^2^] where *P* = (*F*_o_^2^ + 2*F*_c_^2^)/3
*S* = 1.04	(Δ/σ)_max_ = 0.002
7437 reflections	Δρ_max_ = 2.77 e Å^−^^3^
484 parameters	Δρ_min_ = −1.38 e Å^−^^3^

### (5,6-Dimethyl-1,10-phenanthroline)(2-{[2-(diphenylphosphanyl)benzylidene]amino}ethan-1-amine)platinum(II) dinitrate methanol disolvate Special details

**Table:**

Geometry. All esds (except the esd in the dihedral angle between two l.s. planes) are estimated using the full covariance matrix. The cell esds are taken into account individually in the estimation of esds in distances, angles and torsion angles; correlations between esds in cell parameters are only used when they are defined by crystal symmetry. An approximate (isotropic) treatment of cell esds is used for estimating esds involving l.s. planes.
Refinement. Data integration and reduction were undertaken with XDS (Kabsch, 2010). Absorption corrections were applied to the data using SADABS (Krause *et al.*, 2015). The structures were solved by direct methods using SHELXT (Sheldrick, 2015a) and refined with SHELXL2014 (Sheldrick, 2015b) using the Olex^2^ graphical user interface (Dolomanov *et al.*, 2009). All non-hydrogen atoms were located on the electron density maps and refined anisotropically.

### (5,6-Dimethyl-1,10-phenanthroline)(2-{[2-(diphenylphosphanyl)benzylidene]amino}ethan-1-amine)platinum(II) dinitrate methanol disolvate Fractional atomic coordinates and isotropic or equivalent isotropic displacement parameters (Å^2^)

**Table:**

	*x*	*y*	*z*	*U*_iso_*/*U*_eq_	
Pt1	0.26663 (2)	0.87768 (2)	0.61133 (2)	0.02627 (8)	
P1	0.09855 (9)	0.89639 (9)	0.62855 (4)	0.0265 (2)	
N1	0.3115 (3)	0.7812 (3)	0.67692 (13)	0.0290 (7)	
N2	0.3393 (3)	1.0216 (3)	0.68673 (14)	0.0309 (8)	
N3	0.2414 (3)	0.9622 (3)	0.54403 (13)	0.0283 (7)	
N4	0.4185 (3)	0.8543 (3)	0.58758 (14)	0.0292 (7)	
H4A	0.468165	0.870595	0.614330	0.035*	
H4B	0.427217	0.778448	0.577408	0.035*	
C1	0.3119 (3)	0.6634 (4)	0.67141 (17)	0.0326 (9)	
H1	0.292138	0.630952	0.637989	0.039*	
C2	0.3400 (4)	0.5872 (4)	0.71225 (18)	0.0378 (10)	
H2	0.340900	0.504497	0.706610	0.045*	
C3	0.3667 (4)	0.6331 (4)	0.7615 (2)	0.0367 (10)	
H3	0.384959	0.581989	0.790055	0.044*	
C4	0.3666 (3)	0.7555 (4)	0.76877 (16)	0.0316 (9)	
C5	0.3429 (3)	0.8290 (4)	0.72493 (17)	0.0303 (9)	
C6	0.3902 (3)	0.8071 (4)	0.82022 (17)	0.0343 (10)	
C7	0.4071 (4)	0.7244 (4)	0.86580 (18)	0.0437 (11)	
H7A	0.350480	0.665416	0.863801	0.066*	
H7B	0.406364	0.769071	0.898246	0.066*	
H7C	0.475581	0.684777	0.865029	0.066*	
C8	0.3932 (3)	0.9272 (4)	0.82598 (17)	0.0349 (9)	
C9	0.4176 (4)	0.9848 (4)	0.87858 (18)	0.0446 (11)	
H9A	0.494441	0.989675	0.886698	0.067*	
H9B	0.386855	0.937820	0.905257	0.067*	
H9C	0.387298	1.064286	0.877854	0.067*	
C10	0.3755 (3)	1.0031 (4)	0.78090 (17)	0.0324 (9)	
C11	0.3521 (3)	0.9549 (4)	0.73063 (16)	0.0295 (9)	
C12	0.3800 (4)	1.1271 (4)	0.7840 (2)	0.0393 (11)	
H12	0.394391	1.164132	0.816907	0.047*	
C13	0.3638 (4)	1.1941 (4)	0.74024 (18)	0.0385 (10)	
H13	0.365270	1.277623	0.742369	0.046*	
C14	0.3450 (4)	1.1373 (4)	0.6917 (2)	0.0369 (10)	
H14	0.335972	1.184385	0.661237	0.044*	
C15	0.0374 (3)	1.0261 (3)	0.59714 (15)	0.0279 (8)	
C16	−0.0553 (3)	1.0722 (4)	0.61407 (17)	0.0326 (9)	
H16	−0.084325	1.037142	0.643003	0.039*	
C17	−0.1065 (4)	1.1692 (4)	0.58928 (18)	0.0363 (10)	
H17	−0.169825	1.198887	0.601348	0.044*	
C18	−0.0651 (3)	1.2216 (4)	0.54746 (17)	0.0356 (9)	
H18	−0.098841	1.288320	0.531030	0.043*	
C19	0.0261 (3)	1.1762 (4)	0.52962 (16)	0.0333 (9)	
H19	0.053785	1.211959	0.500485	0.040*	
C20	0.0789 (3)	1.0784 (4)	0.55352 (16)	0.0290 (8)	
C21	0.1711 (3)	1.0384 (4)	0.52799 (16)	0.0303 (9)	
H21	0.179904	1.073485	0.495261	0.036*	
C22	0.3266 (3)	0.9377 (4)	0.50960 (16)	0.0339 (9)	
H22A	0.327753	0.999749	0.482685	0.041*	
H22B	0.314209	0.860793	0.492023	0.041*	
C23	0.4301 (3)	0.9360 (4)	0.54336 (16)	0.0325 (9)	
H23A	0.487772	0.908665	0.522936	0.039*	
H23B	0.447790	1.016172	0.556550	0.039*	
C24	0.0141 (3)	0.7732 (3)	0.60688 (15)	0.0295 (9)	
C25	−0.0948 (3)	0.7758 (4)	0.61190 (17)	0.0368 (10)	
H25	−0.125164	0.841140	0.628073	0.044*	
C26	−0.1579 (4)	0.6833 (4)	0.59331 (18)	0.0390 (10)	
H26	−0.231898	0.685773	0.596350	0.047*	
C27	−0.1143 (4)	0.5862 (4)	0.57013 (19)	0.0389 (10)	
H27	−0.158082	0.522578	0.557602	0.047*	
C28	−0.0068 (4)	0.5832 (4)	0.56551 (17)	0.0381 (10)	
H28	0.023360	0.517118	0.549780	0.046*	
C29	0.0576 (3)	0.6761 (4)	0.58369 (15)	0.0306 (9)	
H29	0.131418	0.673391	0.580305	0.037*	
C30	0.0824 (3)	0.9117 (4)	0.69738 (16)	0.0313 (9)	
C31	0.0708 (4)	0.8101 (4)	0.72678 (17)	0.0378 (10)	
H31	0.061421	0.736023	0.709831	0.045*	
C32	0.0728 (4)	0.8163 (5)	0.78048 (18)	0.0457 (12)	
H32	0.065234	0.746749	0.800282	0.055*	
C33	0.0858 (4)	0.9244 (5)	0.80516 (19)	0.0490 (12)	
H33	0.087445	0.928851	0.841980	0.059*	
C34	0.0965 (4)	1.0258 (4)	0.77646 (18)	0.0425 (11)	
H34	0.104560	1.099692	0.793663	0.051*	
C35	0.0955 (3)	1.0206 (4)	0.72290 (17)	0.0357 (9)	
H35	0.103556	1.090507	0.703439	0.043*	
O1	0.5815 (3)	0.8966 (3)	0.67026 (13)	0.0378 (7)	
O2	0.7493 (2)	0.9350 (3)	0.67857 (14)	0.0519 (9)	
O3	0.6696 (3)	0.9005 (4)	0.60208 (14)	0.0547 (9)	
N5	0.6680 (3)	0.9108 (3)	0.65012 (15)	0.0364 (8)	
O4	0.3886 (3)	0.6110 (3)	0.55246 (16)	0.0450 (9)	
O5	0.2954 (3)	0.6488 (3)	0.47932 (13)	0.0441 (8)	
O6	0.2319 (3)	0.5336 (3)	0.53539 (13)	0.0451 (8)	
N6	0.3067 (3)	0.5982 (3)	0.52204 (15)	0.0356 (8)	
O7	0.2720 (3)	0.3786 (3)	0.61778 (15)	0.0431 (10)	
H7	0.269808	0.434623	0.596420	0.065*	
C36	0.1720 (5)	0.3699 (4)	0.6397 (3)	0.0537 (14)	
H36A	0.120403	0.330725	0.614865	0.081*	
H36B	0.146541	0.449053	0.647250	0.081*	
H36C	0.180943	0.324001	0.671986	0.081*	
O8	0.3441 (3)	0.2207 (3)	0.54159 (17)	0.0588 (10)	
H8	0.325735	0.260658	0.566648	0.088*	
C37	0.3934 (4)	0.2952 (5)	0.5070 (2)	0.0545 (13)	
H37A	0.392299	0.257235	0.472879	0.082*	
H37B	0.466893	0.309650	0.520749	0.082*	
H37C	0.355299	0.370287	0.503528	0.082*	

### (5,6-Dimethyl-1,10-phenanthroline)(2-{[2-(diphenylphosphanyl)benzylidene]amino}ethan-1-amine)platinum(II) dinitrate methanol disolvate Atomic displacement parameters (Å^2^)

**Table:**

	*U*^11^	*U*^22^	*U*^33^	*U*^12^	*U*^13^	*U*^23^
Pt1	0.03153 (13)	0.02215 (12)	0.02472 (12)	−0.00001 (5)	0.00038 (7)	0.00103 (5)
P1	0.0315 (6)	0.0235 (5)	0.0241 (5)	−0.0001 (4)	0.0001 (4)	−0.0003 (4)
N1	0.0345 (18)	0.0220 (16)	0.0305 (18)	−0.0020 (13)	0.0023 (14)	−0.0003 (14)
N2	0.0316 (19)	0.0256 (17)	0.0345 (19)	−0.0016 (13)	−0.0027 (14)	0.0004 (15)
N3	0.0321 (18)	0.0221 (16)	0.0303 (18)	−0.0041 (13)	0.0001 (14)	−0.0015 (13)
N4	0.033 (2)	0.0258 (16)	0.0285 (19)	0.0033 (14)	0.0019 (14)	0.0023 (14)
C1	0.040 (2)	0.024 (2)	0.034 (2)	0.0002 (17)	0.0013 (17)	−0.0002 (18)
C2	0.047 (3)	0.026 (2)	0.040 (3)	0.0000 (19)	0.003 (2)	0.004 (2)
C3	0.039 (3)	0.031 (2)	0.039 (3)	−0.0018 (17)	0.002 (2)	0.0086 (18)
C4	0.029 (2)	0.035 (2)	0.031 (2)	−0.0003 (17)	0.0018 (16)	0.0060 (18)
C5	0.029 (2)	0.026 (2)	0.035 (2)	0.0028 (16)	0.0026 (16)	0.0005 (17)
C6	0.031 (2)	0.039 (2)	0.033 (2)	−0.0022 (18)	0.0005 (17)	0.0033 (19)
C7	0.049 (3)	0.048 (3)	0.033 (2)	0.005 (2)	−0.001 (2)	0.010 (2)
C8	0.033 (2)	0.040 (3)	0.032 (2)	0.0008 (18)	0.0015 (17)	−0.0024 (19)
C9	0.058 (3)	0.041 (3)	0.033 (2)	0.000 (2)	−0.004 (2)	−0.002 (2)
C10	0.027 (2)	0.032 (2)	0.038 (2)	0.0039 (16)	0.0011 (17)	−0.0027 (18)
C11	0.027 (2)	0.031 (2)	0.031 (2)	0.0014 (16)	0.0028 (16)	0.0003 (17)
C12	0.040 (3)	0.039 (3)	0.038 (3)	−0.0023 (17)	−0.002 (2)	−0.0085 (18)
C13	0.045 (3)	0.024 (2)	0.045 (3)	0.0005 (17)	−0.001 (2)	−0.0046 (19)
C14	0.043 (3)	0.029 (2)	0.039 (3)	−0.0004 (17)	0.002 (2)	0.0063 (18)
C15	0.035 (2)	0.0237 (19)	0.024 (2)	−0.0011 (15)	−0.0010 (15)	−0.0020 (16)
C16	0.034 (2)	0.027 (2)	0.036 (2)	−0.0020 (17)	−0.0010 (17)	−0.0018 (18)
C17	0.037 (2)	0.027 (2)	0.044 (3)	0.0034 (18)	−0.0027 (19)	−0.0053 (19)
C18	0.046 (3)	0.025 (2)	0.035 (2)	0.0029 (17)	−0.0050 (18)	0.0000 (18)
C19	0.043 (3)	0.025 (2)	0.030 (2)	−0.0020 (17)	−0.0046 (17)	0.0025 (17)
C20	0.034 (2)	0.0229 (19)	0.029 (2)	0.0010 (16)	−0.0044 (16)	−0.0035 (17)
C21	0.036 (2)	0.030 (2)	0.024 (2)	−0.0045 (17)	0.0010 (16)	−0.0012 (16)
C22	0.041 (2)	0.030 (2)	0.031 (2)	0.0015 (17)	0.0030 (17)	0.0031 (17)
C23	0.038 (2)	0.030 (2)	0.030 (2)	0.0000 (17)	0.0033 (17)	0.0047 (17)
C24	0.038 (2)	0.024 (2)	0.025 (2)	−0.0022 (16)	−0.0011 (16)	0.0026 (16)
C25	0.040 (3)	0.032 (2)	0.039 (2)	−0.0016 (18)	0.0023 (18)	−0.0020 (18)
C26	0.037 (2)	0.034 (2)	0.045 (3)	−0.0037 (18)	−0.0029 (19)	0.002 (2)
C27	0.043 (3)	0.030 (2)	0.041 (3)	−0.0047 (19)	−0.008 (2)	0.004 (2)
C28	0.055 (3)	0.025 (2)	0.033 (2)	−0.0012 (19)	−0.005 (2)	0.0002 (19)
C29	0.038 (2)	0.024 (2)	0.029 (2)	−0.0032 (16)	−0.0017 (17)	−0.0012 (17)
C30	0.027 (2)	0.038 (2)	0.028 (2)	0.0019 (17)	0.0010 (16)	0.0016 (19)
C31	0.048 (3)	0.036 (2)	0.029 (2)	−0.0057 (19)	−0.0004 (18)	0.0030 (19)
C32	0.053 (3)	0.050 (3)	0.035 (3)	−0.011 (2)	0.004 (2)	0.003 (2)
C33	0.047 (3)	0.070 (4)	0.030 (2)	0.000 (3)	0.003 (2)	−0.005 (2)
C34	0.043 (3)	0.047 (3)	0.036 (2)	0.004 (2)	−0.0036 (19)	−0.013 (2)
C35	0.033 (2)	0.039 (2)	0.035 (2)	0.0048 (18)	0.0041 (18)	0.0002 (19)
O1	0.0381 (19)	0.0356 (17)	0.0397 (18)	−0.0020 (13)	0.0040 (14)	−0.0003 (14)
O2	0.0343 (18)	0.061 (2)	0.060 (2)	−0.0016 (16)	−0.0001 (16)	−0.0230 (19)
O3	0.051 (2)	0.078 (3)	0.035 (2)	0.0135 (18)	0.0066 (16)	0.0069 (18)
N5	0.042 (2)	0.0278 (18)	0.039 (2)	0.0057 (16)	0.0011 (17)	−0.0012 (17)
O4	0.043 (2)	0.0381 (19)	0.052 (2)	0.0061 (13)	−0.0094 (16)	−0.0032 (15)
O5	0.055 (2)	0.0406 (17)	0.0363 (19)	0.0048 (15)	−0.0001 (15)	0.0075 (15)
O6	0.048 (2)	0.0412 (18)	0.0463 (19)	−0.0014 (15)	0.0075 (15)	−0.0007 (15)
N6	0.043 (2)	0.0304 (18)	0.033 (2)	0.0047 (16)	0.0026 (16)	−0.0061 (16)
O7	0.045 (2)	0.035 (2)	0.050 (2)	0.0022 (12)	0.0050 (18)	0.0111 (13)
C36	0.053 (4)	0.044 (3)	0.062 (4)	0.001 (2)	−0.002 (3)	0.008 (2)
O8	0.068 (3)	0.0364 (19)	0.073 (3)	−0.0019 (17)	0.012 (2)	−0.0042 (18)
C37	0.062 (3)	0.048 (3)	0.055 (3)	0.004 (2)	0.012 (3)	−0.010 (3)

### (5,6-Dimethyl-1,10-phenanthroline)(2-{[2-(diphenylphosphanyl)benzylidene]amino}ethan-1-amine)platinum(II) dinitrate methanol disolvate Geometric parameters (Å, º)

**Table:**

Pt1—P1	2.2290 (13)	C18—C19	1.384 (6)
Pt1—N1	2.052 (3)	C19—H19	0.9500
Pt1—N3	1.984 (3)	C19—C20	1.410 (6)
Pt1—N4	2.092 (4)	C20—C21	1.467 (6)
P1—C15	1.819 (4)	C21—H21	0.9500
P1—C24	1.818 (4)	C22—H22A	0.9900
P1—C30	1.815 (4)	C22—H22B	0.9900
N1—C1	1.345 (5)	C22—C23	1.509 (6)
N1—C5	1.377 (5)	C23—H23A	0.9900
N2—C11	1.360 (5)	C23—H23B	0.9900
N2—C14	1.321 (5)	C24—C25	1.400 (6)
N3—C21	1.283 (5)	C24—C29	1.392 (6)
N3—C22	1.487 (5)	C25—H25	0.9500
N4—H4A	0.9100	C25—C26	1.380 (6)
N4—H4B	0.9100	C26—H26	0.9500
N4—C23	1.488 (5)	C26—C27	1.392 (7)
C1—H1	0.9500	C27—H27	0.9500
C1—C2	1.384 (6)	C27—C28	1.381 (7)
C2—H2	0.9500	C28—H28	0.9500
C2—C3	1.386 (7)	C28—C29	1.388 (6)
C3—H3	0.9500	C29—H29	0.9500
C3—C4	1.402 (6)	C30—C31	1.396 (6)
C4—C5	1.416 (6)	C30—C35	1.402 (6)
C4—C6	1.457 (6)	C31—H31	0.9500
C5—C11	1.440 (6)	C31—C32	1.386 (6)
C6—C7	1.505 (6)	C32—H32	0.9500
C6—C8	1.371 (7)	C32—C33	1.385 (8)
C7—H7A	0.9800	C33—H33	0.9500
C7—H7B	0.9800	C33—C34	1.382 (8)
C7—H7C	0.9800	C34—H34	0.9500
C8—C9	1.513 (6)	C34—C35	1.383 (6)
C8—C10	1.448 (6)	C35—H35	0.9500
C9—H9A	0.9800	O1—N5	1.267 (5)
C9—H9B	0.9800	O2—N5	1.241 (5)
C9—H9C	0.9800	O3—N5	1.248 (5)
C10—C11	1.414 (6)	O4—N6	1.252 (5)
C10—C12	1.410 (6)	O5—N6	1.240 (5)
C12—H12	0.9500	O6—N6	1.272 (5)
C12—C13	1.362 (7)	O7—H7	0.8400
C13—H13	0.9500	O7—C36	1.440 (7)
C13—C14	1.409 (7)	C36—H36A	0.9800
C14—H14	0.9500	C36—H36B	0.9800
C15—C16	1.393 (6)	C36—H36C	0.9800
C15—C20	1.417 (6)	O8—H8	0.8400
C16—H16	0.9500	O8—C37	1.416 (6)
C16—C17	1.403 (6)	C37—H37A	0.9800
C17—H17	0.9500	C37—H37B	0.9800
C17—C18	1.378 (7)	C37—H37C	0.9800
C18—H18	0.9500		
			
N1—Pt1—P1	94.83 (10)	C18—C17—H17	119.9
N1—Pt1—N4	88.92 (13)	C17—C18—H18	120.3
N3—Pt1—P1	92.78 (10)	C17—C18—C19	119.4 (4)
N3—Pt1—N1	171.97 (13)	C19—C18—H18	120.3
N3—Pt1—N4	83.30 (14)	C18—C19—H19	119.1
N4—Pt1—P1	174.15 (10)	C18—C19—C20	121.7 (4)
C15—P1—Pt1	111.42 (14)	C20—C19—H19	119.1
C24—P1—Pt1	114.42 (14)	C15—C20—C21	126.7 (4)
C24—P1—C15	105.63 (19)	C19—C20—C15	118.8 (4)
C30—P1—Pt1	113.62 (14)	C19—C20—C21	114.5 (4)
C30—P1—C15	106.1 (2)	N3—C21—C20	128.6 (4)
C30—P1—C24	105.0 (2)	N3—C21—H21	115.7
C1—N1—Pt1	116.5 (3)	C20—C21—H21	115.7
C1—N1—C5	118.9 (4)	N3—C22—H22A	110.2
C5—N1—Pt1	124.6 (3)	N3—C22—H22B	110.2
C14—N2—C11	118.0 (4)	N3—C22—C23	107.5 (3)
C21—N3—Pt1	131.2 (3)	H22A—C22—H22B	108.5
C21—N3—C22	117.1 (3)	C23—C22—H22A	110.2
C22—N3—Pt1	111.4 (2)	C23—C22—H22B	110.2
Pt1—N4—H4A	110.2	N4—C23—C22	107.9 (3)
Pt1—N4—H4B	110.2	N4—C23—H23A	110.1
H4A—N4—H4B	108.5	N4—C23—H23B	110.1
C23—N4—Pt1	107.8 (3)	C22—C23—H23A	110.1
C23—N4—H4A	110.2	C22—C23—H23B	110.1
C23—N4—H4B	110.2	H23A—C23—H23B	108.4
N1—C1—H1	118.5	C25—C24—P1	121.0 (3)
N1—C1—C2	122.9 (4)	C29—C24—P1	119.7 (3)
C2—C1—H1	118.5	C29—C24—C25	119.3 (4)
C1—C2—H2	120.4	C24—C25—H25	120.1
C1—C2—C3	119.2 (4)	C26—C25—C24	119.9 (4)
C3—C2—H2	120.4	C26—C25—H25	120.1
C2—C3—H3	120.3	C25—C26—H26	119.7
C2—C3—C4	119.5 (4)	C25—C26—C27	120.7 (4)
C4—C3—H3	120.3	C27—C26—H26	119.7
C3—C4—C5	118.6 (4)	C26—C27—H27	120.3
C3—C4—C6	121.2 (4)	C28—C27—C26	119.5 (4)
C5—C4—C6	120.1 (4)	C28—C27—H27	120.3
N1—C5—C4	120.7 (4)	C27—C28—H28	119.8
N1—C5—C11	119.7 (4)	C27—C28—C29	120.5 (4)
C4—C5—C11	119.6 (4)	C29—C28—H28	119.8
C4—C6—C7	117.7 (4)	C24—C29—H29	119.9
C8—C6—C4	120.0 (4)	C28—C29—C24	120.2 (4)
C8—C6—C7	122.3 (4)	C28—C29—H29	119.9
C6—C7—H7A	109.5	C31—C30—P1	118.7 (4)
C6—C7—H7B	109.5	C31—C30—C35	119.1 (4)
C6—C7—H7C	109.5	C35—C30—P1	121.7 (3)
H7A—C7—H7B	109.5	C30—C31—H31	119.7
H7A—C7—H7C	109.5	C32—C31—C30	120.6 (4)
H7B—C7—H7C	109.5	C32—C31—H31	119.7
C6—C8—C9	121.9 (4)	C31—C32—H32	120.1
C6—C8—C10	120.2 (4)	C33—C32—C31	119.7 (5)
C10—C8—C9	117.9 (4)	C33—C32—H32	120.1
C8—C9—H9A	109.5	C32—C33—H33	119.9
C8—C9—H9B	109.5	C34—C33—C32	120.3 (5)
C8—C9—H9C	109.5	C34—C33—H33	119.9
H9A—C9—H9B	109.5	C33—C34—H34	119.7
H9A—C9—H9C	109.5	C33—C34—C35	120.5 (5)
H9B—C9—H9C	109.5	C35—C34—H34	119.7
C11—C10—C8	120.7 (4)	C30—C35—H35	120.1
C12—C10—C8	123.0 (4)	C34—C35—C30	119.8 (4)
C12—C10—C11	116.2 (4)	C34—C35—H35	120.1
N2—C11—C5	117.7 (4)	O2—N5—O1	119.3 (4)
N2—C11—C10	123.3 (4)	O2—N5—O3	121.3 (4)
C10—C11—C5	119.0 (4)	O3—N5—O1	119.4 (4)
C10—C12—H12	119.7	O4—N6—O6	119.6 (4)
C13—C12—C10	120.5 (4)	O5—N6—O4	121.7 (4)
C13—C12—H12	119.7	O5—N6—O6	118.7 (4)
C12—C13—H13	120.6	C36—O7—H7	109.5
C12—C13—C14	118.8 (4)	O7—C36—H36A	109.5
C14—C13—H13	120.6	O7—C36—H36B	109.5
N2—C14—C13	123.0 (4)	O7—C36—H36C	109.5
N2—C14—H14	118.5	H36A—C36—H36B	109.5
C13—C14—H14	118.5	H36A—C36—H36C	109.5
C16—C15—P1	120.1 (3)	H36B—C36—H36C	109.5
C16—C15—C20	118.6 (4)	C37—O8—H8	109.5
C20—C15—P1	121.2 (3)	O8—C37—H37A	109.5
C15—C16—H16	119.3	O8—C37—H37B	109.5
C15—C16—C17	121.4 (4)	O8—C37—H37C	109.5
C17—C16—H16	119.3	H37A—C37—H37B	109.5
C16—C17—H17	119.9	H37A—C37—H37C	109.5
C18—C17—C16	120.1 (4)	H37B—C37—H37C	109.5
			
Pt1—P1—C15—C16	−162.0 (3)	C8—C10—C12—C13	179.3 (4)
Pt1—P1—C15—C20	21.5 (4)	C9—C8—C10—C11	−179.8 (4)
Pt1—P1—C24—C25	−175.8 (3)	C9—C8—C10—C12	0.0 (6)
Pt1—P1—C24—C29	2.5 (4)	C10—C12—C13—C14	−1.4 (7)
Pt1—P1—C30—C31	−89.2 (3)	C11—N2—C14—C13	0.1 (7)
Pt1—P1—C30—C35	82.2 (4)	C11—C10—C12—C13	−1.0 (7)
Pt1—N1—C1—C2	−179.7 (3)	C12—C10—C11—N2	3.2 (6)
Pt1—N1—C5—C4	176.2 (3)	C12—C10—C11—C5	−178.0 (4)
Pt1—N1—C5—C11	−3.9 (5)	C12—C13—C14—N2	1.9 (7)
Pt1—N3—C21—C20	−6.9 (6)	C14—N2—C11—C5	178.4 (4)
Pt1—N3—C22—C23	−39.7 (4)	C14—N2—C11—C10	−2.8 (6)
Pt1—N4—C23—C22	−39.0 (4)	C15—P1—C24—C25	−52.9 (4)
P1—C15—C16—C17	−177.5 (3)	C15—P1—C24—C29	125.4 (3)
P1—C15—C20—C19	177.8 (3)	C15—P1—C30—C31	148.1 (3)
P1—C15—C20—C21	0.6 (6)	C15—P1—C30—C35	−40.6 (4)
P1—C24—C25—C26	177.5 (3)	C15—C16—C17—C18	−0.4 (7)
P1—C24—C29—C28	−178.0 (3)	C15—C20—C21—N3	−12.1 (7)
P1—C30—C31—C32	171.1 (4)	C16—C15—C20—C19	1.2 (6)
P1—C30—C35—C34	−171.4 (3)	C16—C15—C20—C21	−176.0 (4)
N1—C1—C2—C3	1.5 (7)	C16—C17—C18—C19	1.2 (6)
N1—C5—C11—N2	−6.5 (5)	C17—C18—C19—C20	−0.8 (6)
N1—C5—C11—C10	174.6 (4)	C18—C19—C20—C15	−0.4 (6)
N3—C22—C23—N4	51.6 (4)	C18—C19—C20—C21	177.1 (4)
C1—N1—C5—C4	−5.4 (6)	C19—C20—C21—N3	170.6 (4)
C1—N1—C5—C11	174.5 (4)	C20—C15—C16—C17	−0.8 (6)
C1—C2—C3—C4	−1.1 (7)	C21—N3—C22—C23	135.1 (4)
C2—C3—C4—C5	−2.4 (7)	C22—N3—C21—C20	179.5 (4)
C2—C3—C4—C6	177.6 (4)	C24—P1—C15—C16	73.2 (4)
C3—C4—C5—N1	5.7 (6)	C24—P1—C15—C20	−103.3 (3)
C3—C4—C5—C11	−174.1 (4)	C24—P1—C30—C31	36.5 (4)
C3—C4—C6—C7	−4.2 (6)	C24—P1—C30—C35	−152.1 (4)
C3—C4—C6—C8	177.7 (4)	C24—C25—C26—C27	0.9 (7)
C4—C5—C11—N2	173.4 (4)	C25—C24—C29—C28	0.4 (6)
C4—C5—C11—C10	−5.5 (5)	C25—C26—C27—C28	−0.4 (7)
C4—C6—C8—C9	−179.9 (4)	C26—C27—C28—C29	−0.1 (7)
C4—C6—C8—C10	−1.5 (6)	C27—C28—C29—C24	0.1 (7)
C5—N1—C1—C2	1.8 (6)	C29—C24—C25—C26	−0.8 (6)
C5—C4—C6—C7	175.8 (4)	C30—P1—C15—C16	−37.9 (4)
C5—C4—C6—C8	−2.3 (6)	C30—P1—C15—C20	145.6 (3)
C6—C4—C5—N1	−174.3 (4)	C30—P1—C24—C25	59.0 (4)
C6—C4—C5—C11	5.8 (6)	C30—P1—C24—C29	−122.7 (3)
C6—C8—C10—C11	1.8 (6)	C30—C31—C32—C33	0.4 (7)
C6—C8—C10—C12	−178.5 (4)	C31—C30—C35—C34	−0.1 (6)
C7—C6—C8—C9	2.1 (6)	C31—C32—C33—C34	0.2 (7)
C7—C6—C8—C10	−179.5 (4)	C32—C33—C34—C35	−0.7 (7)
C8—C10—C11—N2	−177.1 (4)	C33—C34—C35—C30	0.7 (7)
C8—C10—C11—C5	1.8 (6)	C35—C30—C31—C32	−0.4 (7)

### (5,6-Dimethyl-1,10-phenanthroline)(2-{[2-(diphenylphosphanyl)benzylidene]amino}ethan-1-amine)platinum(II) dinitrate methanol disolvate Hydrogen-bond geometry (Å, º)

**Table:**

*D*—H···*A*	*D*—H	H···*A*	*D*···*A*	*D*—H···*A*
N4—H4*A*···O1	0.91	1.96	2.872 (5)	176
N4—H4*B*···O4	0.91	2.05	2.920 (5)	159
O7—H7···O6	0.84	1.96	2.771 (5)	162
O8—H8···O7	0.84	2.04	2.872 (5)	170
C3—H3···O1^i^	0.95	2.36	3.244 (6)	154
C3—H3···O2^i^	0.95	2.57	3.168 (6)	121
C9—H9*A*···O4^ii^	0.98	2.48	3.235 (6)	134
C9—H9*B*···O5^iii^	0.98	2.53	3.494 (6)	170
C14—H14···O7^iv^	0.95	2.57	3.417 (6)	149
C18—H18···O5^v^	0.95	2.58	3.288 (6)	131
C22—H22*A*···O3^vi^	0.99	2.47	3.422 (6)	162
C22—H22*B*···O5	0.99	2.44	3.385 (6)	160
C25—H25···O2^vii^	0.95	2.40	3.282 (5)	154
C27—H27···O6^viii^	0.95	2.58	3.278 (6)	131
C29—H29···O6	0.95	2.40	3.094 (5)	130

Symmetry codes: (i) −*x*+1, *y*−1/2, −*z*+3/2; (ii) −*x*+1, *y*+1/2, −*z*+3/2; (iii) *x*, −*y*+3/2, *z*+1/2; (iv) *x*, *y*+1, *z*; (v) −*x*, −*y*+2, −*z*+1; (vi) −*x*+1, −*y*+2, −*z*+1; (vii) *x*−1, *y*, *z*; (viii) −*x*, −*y*+1, −*z*+1.

## References

[bb1] Brodie, C. R., Collins, J. G. & Aldrich-Wright, J. R. (2004). *Dalton Trans.* pp. 1145–1152. 10.1039/b316511f 15252653

[bb2] Brodie, C. R., Turner, P., Wheate, N. J. & Aldrich-Wright, J. R. (2006). *Acta Cryst.* E**62**, m3137–m3139.

[bb3] Bushnell, G. W., Dixon, K. R. & Khan, M. A. (1974). *Can. J. Chem.* **52**, 1367–1376.

[bb4] Cowieson, N. P., Aragao, D., Clift, M., Ericsson, D. J., Gee, C., Harrop, S. J., Mudie, N., Panjikar, S., Price, J. R., Riboldi-Tunnicliffe, A., Williamson, R. & Caradoc-Davies, T. (2015). *J. Synchrotron Rad.* **22**, 187–190. 10.1107/S1600577514021717 PMC4294030 25537608

[bb5] Dolomanov, O. V., Bourhis, L. J., Gildea, R. J., Howard, J. A. K. & Puschmann, H. (2009). *J. Appl. Cryst.* **42**, 339–341.

[bb6] Ellis, L. T. & Hambley, T. W. (1994). *Acta Cryst.* C**50**, 1888–1889.

[bb7] Fanizzi, F. P., Lanfranchi, M., Natile, G. & Tiripicchio, A. (1994). *Inorg. Chem.* **33**, 3331–3339. 10.1021/ic960125y 11666514

[bb8] Fanizzi, F. P., Maresca, L., Natile, G., Lanfranchi, M., Tiripicchio, A. & Pacchioni, G. (1992). *J. Chem. Soc. Chem. Commun.* pp. 333–338.

[bb9] Fanizzi, F. P., Margiotta, N., Lanfranchi, M., Tiripicchio, A., Pacchioni, G. & Natile, G. (2004). *Eur. J. Inorg. Chem.* pp. 1705–1713.

[bb10] Groom, C. R., Bruno, I. J., Lightfoot, M. P. & Ward, S. C. (2016). *Acta Cryst.* B**72**, 171–179. 10.1107/S2052520616003954 PMC4822653 27048719

[bb11] Kabsch, W. (1993). *J. Appl. Cryst.* **26**, 795–800.

[bb12] Kabsch, W. (2010). *Acta Cryst.* D**66**, 133–144. 10.1107/S0907444909047374 PMC2815666 20124693

[bb13] Kato, M. & Takahashi, J. (1999). *Acta Cryst.* C**55**, 1809–1812.

[bb14] Kemp, S., Wheate, N. J., Buck, D. P., Nikac, M., Collins, J. G. & Aldrich-Wright, J. R. (2007). *J. Inorg. Biochem.* **101**, 1049–1058. 10.1016/j.jinorgbio.2007.04.009 17544512

[bb15] Khoury, A., Sakoff, J. A., Gilbert, J., Scott, K. F., Karan, S., Gordon, C. P. & Aldrich-Wright, J. R. (2022). *Pharmaceutics*, **14**, 787. 10.3390/pharmaceutics14040787 PMC9029360 35456621

[bb16] Krause, L., Herbst-Irmer, R., Sheldrick, G. M. & Stalke, D. (2015). *J. Appl. Cryst.* **48**, 3–10. 10.1107/S1600576714022985 PMC4453166 26089746

[bb17] McPhillips, T. M., McPhillips, S. E., Chiu, H.-J., Cohen, A. E., Deacon, A. M., Ellis, P. J., Garman, E., Gonzalez, A., Sauter, N. K., Phizackerley, R. P., Soltis, S. M. & Kuhn, P. (2002). *J. Synchrotron Rad.* **9**, 401–406. 10.1107/s0909049502015170 12409628

[bb18] Sheldrick, G. M. (2015*a*). *Acta Cryst.* A**71**, 3–8.

[bb19] Sheldrick, G. M. (2015*b*). *Acta Cryst.* C**71**, 3–8.

[bb20] Spek, A. L. (2020). *Acta Cryst.* E**76**, 1–11. 10.1107/S2056989019016244 PMC6944088 31921444

[bb21] Usón, R., Forniés, J., Tomás, M., Martínez, F., Casas, J. M. & Fortuño, C. (1995). *Inorg. Chim. Acta*, **235**, 51–60.

